# Calpain 3 deficiency affects SERCA expression and function in the skeletal muscle

**DOI:** 10.1017/erm.2016.9

**Published:** 2016-04-08

**Authors:** Ivan Toral-Ojeda, Garazi Aldanondo, Jaione Lasa-Elgarresta, Haizpea Lasa-Fernández, Roberto Fernández-Torrón, Adolfo López de Munain, Ainara Vallejo-Illarramendi

**Affiliations:** 1 Neuroscience Area, Biodonostia Research Institute, San Sebastian, Spain; 2 CIBERNED, Instituto de Salud Carlos III, Madrid, Spain; 3 Department of Neuroscience, University of the Basque Country, San Sebastian, Spain; 4 Department of Neurology, Hospital Universitario Donostia, San Sebastian, Spain

## Abstract

Limb-girdle muscular dystrophy type 2A (LGMD2A) is a form of muscular dystrophy caused by mutations in calpain 3 (CAPN3). Several studies have implicated Ca^2+^ dysregulation as an underlying event in several muscular dystrophies, including LGMD2A. In this study we used mouse and human myotube cultures, and muscle biopsies in order to determine whether dysfunction of sarco/endoplasmatic Ca^2+^-ATPase (SERCA) is involved in the pathology of this disease. In CAPN3-deficient myotubes, we found decreased levels of SERCA 1 and 2 proteins, while mRNA levels remained comparable with control myotubes. Also, we found a significant reduction in SERCA function that resulted in impairment of Ca^2+^ homeostasis, and elevated basal intracellular [Ca^2+^] in human myotubes. Furthermore, small Ankyrin 1 (sAnk1), a SERCA1-binding protein that is involved in sarcoplasmic reticulum integrity, was also diminished in CAPN3-deficient fibres. Interestingly, SERCA2 protein was patently reduced in muscles from LGMD2A patients, while it was normally expressed in other forms of muscular dystrophy. Thus, analysis of SERCA2 expression may prove useful for diagnostic purposes as a potential indicator of CAPN3 deficiency in muscle biopsies. Altogether, our results indicate that CAPN3 deficiency leads to degradation of SERCA proteins and Ca^2+^ dysregulation in the skeletal muscle. While further studies are needed in order to elucidate the specific contribution of SERCA towards muscle degeneration in LGMD2A, this study constitutes a reasonable foundation for the development of therapeutic approaches targeting SERCA1, SERCA2 or sAnk1.

## Introduction

Limb-girdle muscular dystrophy type 2A (LGMD2A) is a neuromuscular disease caused by mutations in the gene encoding calpain 3 (CAPN3), a nonlysosomal cysteine protease necessary for normal muscle function and regeneration (Refs 1, 2). The exact pathogenic mechanism that leads mutations in *CAPN3* to cause muscular dystrophy remains unclear but accumulated evidence support a multifunctional role of CAPN3 in muscle homeostasis. Moreover, an efficient therapy is not currently available for LGMD2A patients. Previous studies performed on *Capn3* knockout mice describe a reduced expression of the ryanodine receptor type 1 (RyR1) and reduced Ca^2+^ release from the sarcoplasmic reticulum (SR) to cytoplasm (Refs 3, 4), suggesting that dysregulation of Ca^2+^ homeostasis plays a role in the pathogenic mechanisms involved in this form of muscular dystrophy (Ref. 5). Reinforcing this line of evidence, we have recently contributed to a study demonstrating a reduction of RyR1 expression and βCamKII signalling in LGMD2A muscles (Ref. 6). Here, we sought to characterise more in detail the pathway leading to abnormal Ca^2+^ regulation in CAPN3-deficient muscle fibres. In particular, we wanted to analyse the sarco/endoplasmic reticulum Ca^2+^ ATPases (SERCAs), which mediate Ca^2+^ uptake into the SR and enable muscular relaxation (Ref. 7). Interaction between Capn3 with SERCA1 has been previously shown by co-immunoprecipitation and pull-down assays (Ref. 8). Moreover, *Capn3* knockout myotubes display lower SR Ca^2+^ levels as well as a reduced response to the specific SERCA inhibitor cyclopiazonic acid (Ref. 3). Thus, we hypothesised that presence of CAPN3 is required for appropriate SERCA function. In this study, we have focused on the main SERCA proteins expressed in the skeletal muscle that are SERCA1 and SERCA2a, the latest being a SERCA2 isoform specifically expressed in cardiac and slow muscle fibres (Refs 9, 10).

## Materials and methods

### Reagents

Antibodies were obtained from the following sources: SPA-Calpain-3 polyclonal antibody (pAb) (Triple Point Biologics); goat anti-Calpain-3 (pIS2C) pAb (Cosmo Bio Co., LTD); 12A2-Calpain3 monoclonal antibody (mAb) (NCL-CALP-12A2) and Dysferlin mAb (NCL-Hamlet, Leica Biosystems); SERCA2 mAb (sc-376235, Santa Cruz Biotechnology); SERCA2a ATPase pAb (A010-20, Badrilla Ltd.); Ryanodine Receptor mAb (MA3-925) and SERCA1 ATPase mAb (MA3-912, Affinity BioReagents); DHPRalpha2 subunit mAb (ab2864, Abcam); ANK1 pAb (ARP42566_T100, Aviva Systems Biology); Ubiquitin mAb (U0508), TRPC1 pAb (T8276) and Actin pAb (A2066, Sigma-Aldrich); SUMO-1 mAb (#4940S, Cell Signaling); Parvalbumin mAb (#MAB1572, Millipore); Myosin Heavy Chain-CFS mAb (IC4470F, R&D Systems); Myosin Heavy Chain mAbs (A4.1025, A4.840) and Dystrophin mAb (MANDYS1) (Developmental Studies Hybridoma Bank). Lentiviral particles were produced from plasmid DNAs TRCN0000030674 (mouse Capn3-shRNA), TRCN0000003494 (human CAPN3-shRNA) and SHC002 (NS-shRNA) (Sigma-Aldrich).

### Cell cultures

C2C12 mouse myoblasts (ATCC) and LHCN-M2 immortalised human myoblasts (Ref. 11) were grown and differentiated as previously described (Ref. 12). C2C12 myoblasts at 70% confluence were treated for 7 h with shRNA-lentivirus (MOI 10). On the next day, C2C12 infected myoblasts were differentiated for 7 days. Puromycin was added after 5 days of differentiation at 2 µg/ml, to select C2C12 myotubes infected with lentivirus. LHCN-M2 myoblasts were infected at MOI 5 for 24 h and allowed it to grow in Skeletal Growth Medium (C-23160, Promocell) for 2 days before selection with puromycin (1 µg/ml for 7 days). To induce differentiation, nonserum medium was added to myoblasts grown to confluence. Mature myotubes were obtained after 9–14 days in differentiation medium.

### Human muscles

Open muscle biopsies from nine dystrophic patients and four control subjects with non-neuromuscular diseases were obtained through the Department of Neurology of Donostia Hospital using institutionally approved protocols. Clinical information from the subjects is shown in Table 1.

**Table 1.** Clinical characteristics of human muscle biopsies

Code	Diagnosis	Age	Sex	Muscle	GMW scale	Mutation
C1	Control	27	M	Gluteus		
C2	Control	14	F	Dorsal (T4–T11)		
C3	Control	46	M	Vastus lateralis		
C4	Control	64	F	Gluteus		
P1	LGMD2A	28	M	Deltoid	II	p.(Lys254Glu)/p.(Pro637Hisfs*25)
P2	LGMD2A	16	F	Biceps	II–III	p.(Arg490Trp)/p.(Gly691Trpfs*7)
P3	LGMD2A	51	M	Deltoid	IV	p.(Ser479Gly)/p.(Asp665Leufs*18)
P4	LGMD2A	47	F	Deltoid	VII	p.(Arg489Trp)/p.(Arg788Serfs*14)
DMD	Duchenne	15	M	Deltoid		
LG2B	LGMD2B	54	M	Tibialis Anterior		
FSH2	FSH2	36	M	Deltoid		
DM1(m)	DM1	56	M	Deltoid		
DM1(f)	DM1	32	F	Biceps		

### Western blotting and immunoprecipitation

Proteins from cells and muscles were extracted directly in SDS sample buffer and resolved in precast 4–20% gradient SDS–PAGE gels (Bio-Rad). Proteins were transferred onto nitrocellulose membranes, blocked with 5% nonfat milk and incubated with primary antibodies overnight at 4°C. Membranes were incubated with horseradish peroxidase-conjugated secondary antibodies (Santa Cruz), followed by enhanced chemiluminescence reagent (ECL Prime, Amersham) and signals were detected by autoradiographic film exposure. Protein band densitometry was analysed using the NIH Image J software and MyHC immunoreactivity was used to normalise SERCA1/2 signals. For immunoprecipitation assays, we used a muscle biopsy from a healthy donor (*vastus lateralis*) and LHCN-M2 myotubes. Samples were homogenated in modified RIPA assay buffer (10 mm Tris, 100 mm NaCl, 1 mm EDTA, 1 mm EGTA, 20 mm Na_4_P_2_O_7_, 1% Triton X-100, 0.5% sodium deoxycholate, 0.1% SDS, 10% glycerol, pH 7.5) and 1× Halt Protease & Phosphatase Inhibitor cocktail (Thermo Scientific). Protein lysates (500 µg) were incubated with protein G sepharose (GE Healthcare) in 500 µl of RIPA buffer at 4°C for 1 h, to minimise nonspecific binding. After centrifugation, supernatants were incubated with specific antibodies against SERCA1, SERCA2 and CAPN3 (pIS2C) overnight at 4°C. Subsequently, protein G sepharose was added to each sample and reacted at 4°C for 1 h. After washing with RIPA buffer, immunoprecipitated molecules were directly boiled in SDS sample buffer. Normal Mouse and Goat IgGs (Santa Cruz Biotechnology) were used as negative controls for immunoprecipitation. Uncropped western blot scans are shown in supplementary data (Supplementary Figs S1–S6).

### SERCA activity assay

Ca^2+^-dependent SERCA ATPase activity was determined spectrophotometrically in C2C12 and LHCN-M2 myotube homogenates, as previously described with minor modifications (Refs 13, 14). Decline of NADH absorbance at 340 nm was used to analyse the rate of steady-state ATP hydrolysis. Protein homogenates (150–400 µg) were added to 90 µl of reaction buffer (200 mM KCl, 20 mm HEPES, pH 7.0, 15 mm MgCl_2_, 10 mm NaN_3_, 10 mm phosphoenolpyruvate, 5 mm ATP, 1 mm EGTA) with protease inhibitors (Complete mini EDTA free, Roche). Immediately before starting the reaction, 2 µl of PK/LDH at 18 U/ml (P0294, Sigma) and 0.53 µl of 0.19 mm calcimycin (C7522, Sigma) were added to the reaction buffer and this was used as a blank. Reaction was started by addition of 2 µl of 100 mm NADH (N4505, Sigma) and three measurements were taken in 1-min intervals using a NanoDrop ND-1000 to determine basal activity. Maximal SERCA activity was determined by adding 4 µl of 20 mm CaCl_2_ and measurements were recorded for 5 min in 30 s intervals. 1 mm thapsigargin (Sigma) was added to inhibit SERCA activity and measurements were recorded for the next 5 min. The rate of ATP hydrolysis (mm of ATP/mg of protein/min) was estimated from the equation ΔOD_340_/(Δ*t* • ε • *L* • *P*), where ΔOD_340_/Δ*t* is the decrease in absorbance at 340 nm during 5 min and is estimated by linear regression analysis of plotting OD_340_ versus time (min); ε is the NADH extinction coefficient (6.22 • 10^−3^ ml/mm/cm); *L* is the optical pathlength (0.1 cm); and *P* is the amount of protein in mg/ml. Specific SERCA ATPase activity was calculated by subtracting ATPase activity in the presence of thapsigargin from the maximal ATPase activity.

### Calcium imaging

Myotubes grown on glass coverslips were loaded with 4 µm Fura 2-AM and 0.02% pluronic acid in culture medium for 30 min at 37°C. Cells were left in Ringer buffer (125 mm NaCl, 5 mm KCl, 1.2 mm MgSO_4_, 6 mm glucose, 2 mm CaCl_2_ and 25 mm HEPES, pH 7.4) for 20 min at room temperature (RT) to remove nonhydrolysed fluorophore and complete de-esterification of the dye. Experiments were performed under continuous perfusion (2 ml/min) with ringer buffer using an ECLIPSE Ti-S/L100 microscope (Nikon) equipped with a 20× S-Fluor objective and attached to a lambda-DG4 illumination system. Image acquisition was performed using an Orca-Flash2.8 camera (Hammamatsu) with the NisElements-AR software. Ca^2+^ transients were elicited by local addition of 50 or 130 mm KCl in Ca^2+^-free Ringer buffer (80 mm NaCl, 1.2 mm MgSO_4_, 6 mm glucose, 1 mm EGTA and 25 mm HEPES, pH 7.4). Intracellular calcium concentration was estimated by the ratio of Fura 2-AM fluorescence intensities at excitation wavelength 340 and 380 nm. Tau values obtained by fitting the decay of the Ca^2+^ transients with an exponential function were used as a measure of the SERCA ATPase Ca^2+^ clearance activity.

### Immunohistochemistry

Briefly, 10-μm cryostat sections from muscle biopsies were fixed 10 min in pre-cooled acetone, air dried for 5 min and preincubated with 2% horse serum, 5% bovine serum albumin, 0.5% Triton X-100 in PBS for 1 h at RT. CAPN3 pIS2C (1:200), SERCA1 (1:500), SERCA2a ATPase (1:100), ANK1 (1:100) primary antibodies were incubated overnight at 4°C. After washing, Alexa Fluor 488, 555 or 647-conjugated secondary antibodies (1:400; Molecular Probes) were added for 1 h and slides were washed and mounted with Prolong Gold Antifade Reagent with DAPI (Life Technologies). High-resolution images were acquired using a LSM510 Meta confocal microscope (Carl Zeiss, Jena, Germany).

### In situ proximity ligation assay (in situ PLA)

Duolink II Red Fluorescence Kit (Olink Bioscience) was used following manufacturer instructions. Briefly, 10-μm cryosections from a control muscle biopsy were blocked and incubated in the following primary antibodies: pIS2C (1:200), 12A2-Calpain3 (1:50), SERCA1 (1:500), SERCA2a (1:100) and ANK1 (1:100), using the same conditions as for immunohistochemistry. Sections were incubated with oligonucleotide strand conjugated secondary antibodies (MINUS and PLUS PLA probes, Olink Bioscience). After the ligation and amplification steps, sections were incubated with FITC-conjugated Myosin Heavy Chain-CFS antibody (1:50) and mounted with Duolink II Mounting Medium with DAPI (Sigma).

### RT–qPCR

CAPN3 and SERCA expression was quantified using cDNA synthesised from DNase-treated RNA obtained from control and CAPN3 knockdown LHCN-M2 myotubes. qPCR was performed and analysed with the 7900HT Real-Time PCR System (Applied Biosystems), using SyberGreen master mix as previously described (Ref. 15). Technical triplicate measurements were performed in three different cultures, and the results were normalised to a normalisation factor based on the geometric mean of four reference genes: creatine kinase, DHPR, dystrophin and HPRT1. The primer sequences used are shown in Supplementary Table S1.

### Creatine kinase (CK) activity

Total CK activity has been previously used to determine levels of myotube differentiation and maturation (Ref. 16). CK activity was determined with the colorimetric kit CK-NAC (Thermo Scientific) in myotubes lysed with 0.1% Triton solution. CK activity (U/l) was normalised to total protein (μg).

### Study approval

The study was approved by the Ethical Committee Board of Donostia Hospital and informed consent was obtained in accordance with the Declaration of Helsinki.

### Statistics

Data are presented as mean ± SEM. *P* values were defined using Student's *t*-test for paired or nonpaired comparisons. *P* ≤ 0.05 was considered statistically significant.

## Results

### Analysis of SERCA expression and function in C2C12 mouse myotubes

**Figure 1.**
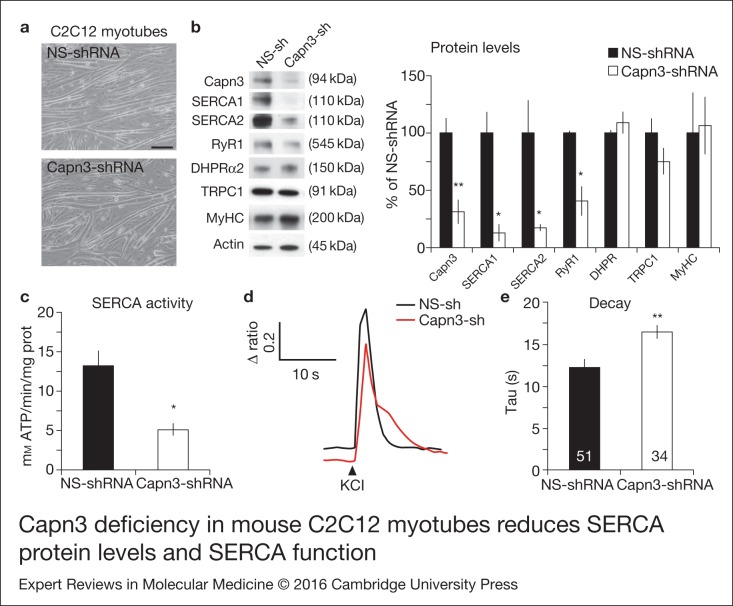
Capn3 deficiency in mouse C2C12 myotubes reduces SERCA protein levels and SERCA function. (a) C2C12 myotubes treated with NS or Capn3 shRNAs and differentiated for 7 days. Scale bar = 100  μm (b) Western blot analysis showing significant decrease of Capn3 (SPA antibody), SERCA1, SERCA2 and RyR1 levels in Capn3 knockdown myotubes, compared with controls (*P < 0.05; **P < 0.01). Total levels of DHPR, TRPC1, MyHC and actin are not significantly changed. *N* = 3 independent experiments run on the same gel. (c) SERCA-specific ATPase activity determined in homogenates from C2C12 myotubes. Capn3-deficient myotubes show a significant reduction of SERCA activity compared with NS-controls (*n* = 3, *P < 0.05). (d and e) Ca^2+^ imaging of C2C12 myotubes loaded with Fura2-AM shows delayed Ca^2+^ clearance from the cytosol in Capn3 knockdown myotubes. (d) Two representative traces of changes in Fura2-AM fluorescence ratios (*F*_340_/*F*_380_) from Capn3-shRNA and NS-shRNA treated myotubes. Ca^2+^ transients were elicited by local stimulation with KCl 50 mm in the absence of extracellular Ca^2+^. (e) Tau, the time constant of the Ca^2+^ transient decay phase in seconds (s), is significantly increased in Capn3-deficient myotubes. **P < 0.01, *n*= total number of myotubes recorded from six different experiments are shown in the graph.

To better understand the role of CAPN3 in the Ca^2+^ homeostasis of skeletal muscle, we used lentiviral shRNAs to knock down Capn3 expression in mouse C2C12 myotubes. No obvious morphological differences were found between Capn3 knockdown C2C12 myotubes and myotubes treated with a nonsilencing (NS) shRNA lentivirus (Fig. 1a, Fig. S7A). Infection with Capn3-shRNA lentivirus caused a 70% reduction of Capn3 protein in C2C12 differentiated myotubes, as assessed by Western blot (Fig. 1b, *P* ≤ 0.01). Capn3 knockdown resulted in a significant decrease in the expression levels of several key Ca^2+^-handling proteins, such as RyR1 (40.9 ± 13.1%), SERCA1 (13 ± 7.9%) and SERCA2 (17.5 ± 2.9%, *P* ≤ 0.05) compared with controls (100%, *n* ≥ 3). In contrast, levels of the dihydropyridine receptor DHPR alpha2 subunit or the mechanosensitive voltage-independent Ca^2+^ channel TRPC-1 were found unchanged. Therefore, our findings support our hypothesis of Capn3 deficiency causing a reduction of SERCA1/2 expression levels, which in turn may lead to a general dysregulation of Ca^2+^ homeostasis.

To further investigate the functional significance of this finding, we analysed SERCA-specific ATPase activity in homogenates from C2C12 myotubes and found a 61% decline in SERCA activity in Capn3 knockdown myotubes (5.13 ± 0.75 mm ATP/min/mg protein) compared with NS controls (13.13 ± 1.95 mm ATP/min/mg protein; *P* < 0.05, Fig. 1c). Next, we sought to verify reduction of SERCA activity in myotube cultures by live Ca^2+^ imaging. In skeletal muscle fibres, cytosolic Ca^2+^ clearance is highly dependent on SERCA activity and, thus, the decay kinetics of Ca^2+^ transients is a good approximation of the efficiency of SERCA-dependent Ca^2+^ uptake (Ref. 17). Control and Capn3 knockdown myotubes were loaded with Fura2-AM and locally stimulated with 50 mm KCl in the absence of extracellular Ca^2+^ and the decay phase of Ca^2+^ transients was analysed as an indicator of SERCA activity (Fig. 1d). Predictably, we found that the Ca^2+^ clearance time is significantly increased in Capn3 knockdown myotubes (16.46 ± 0.80 s) compared with NS controls (12.31 ± 0.99 s, *P* < 0.005). No differences in basal intracellular Ca^2+^ levels were found between control and Capn3-deficient myotubes (Fig. S8), which is in agreement with previous data using primary myotubes from Capn3 knockout mice (Ref. 3).

### Analysis of SERCA expression and function in human myotubes

**Figure 2.**
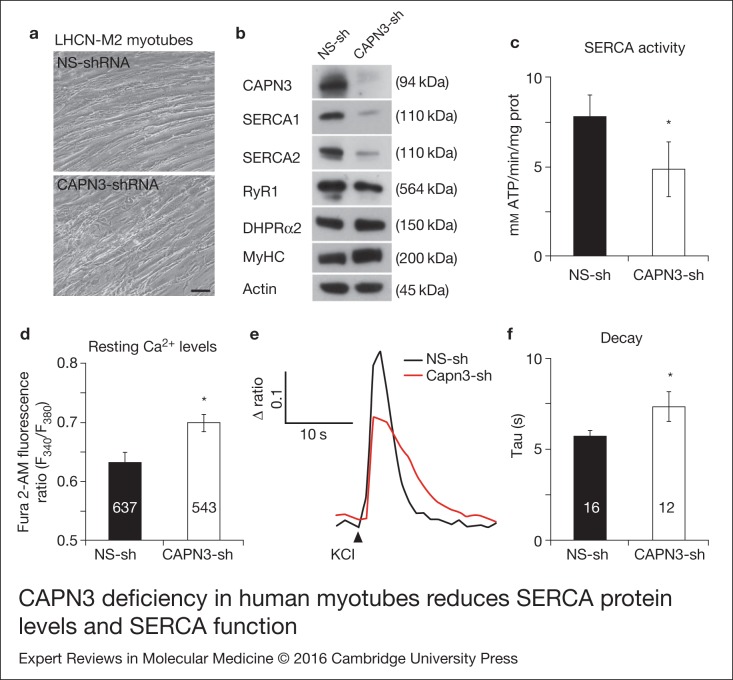
CAPN3 deficiency in human myotubes reduces SERCA protein levels and SERCA function. (a) LHCN-M2 myoblasts treated with control NS-shRNA or CAPN3-shRNAs and differentiated for 9 days. Scale bar = 50 μm. (b) Representative Western blot analysis showing decrease of CAPN3 (12A2 mAb), SERCA1, SERCA2 and RyR1 protein levels in CAPN3-sh treated myotubes compared with controls. DHPRα2, MyHC and actin levels remain unaltered. (c) SERCA-specific ATPase activity determined in homogenates from LHCN-M2 myotubes. CAPN3-deficient myotubes show a significant reduction of SERCA activity compared with NS-controls (*n* = 3, *P < 0.05). D-F) Ca^2+^ imaging of LHCN-M2 myotubes loaded with Fura2-AM shows increased cytosolic [Ca^2+^] and delayed Ca^2+^ clearance from the cytosol in CAPN3 knockdown myotubes. (d) Resting cytosolic [Ca^2+^] was measured in the presence of 2 mm Ca^2+^ at 37°C. CAPN3-deficient human myotubes show significantly increased resting cytosolic [Ca^2+^] compared with controls (*P < 0.05; *n* = 10 experiments). Total numbers of myotubes recorded are shown in the graph. (e) Two representative traces of changes in Fura2-AM fluorescence ratios (*F*_340_/*F*_380_) from CAPN3-shRNA and NS-shRNA treated myotubes. Ca^2+^ transients were elicited by local stimulation with KCl 130 mm in the absence of extracellular Ca^2+^. (f) Tau, the time constant of the Ca^2+^ transient decay phase in seconds (s), is significantly increased in CAPN3-deficient myotubes. *P < 0.05, *n*= total number of myotubes recorded from three different experiments are shown in the graph.

Next, we used the human myoblast line LHCN-M2 (Ref. 11) to study the effect of CAPN3 knockdown on SERCA1/2 expression. LHCN-M2 myoblasts were treated with a human specific CAPN3-shRNA or the NS-shRNA, and allowed to differentiate for 9–14 days. We did not observe any difference in proliferation or differentiation between myoblasts treated with the different shRNAs (Fig. 2a, Fig. S7). CAPN3-shRNA treated myotubes (CAPN3-sh) showed a clear reduction of CAPN3 protein levels compared with controls (NS-sh), as determined by Western blot analysis. Similar to C2C12 myotubes, knocking down CAPN3 in human myotubes resulted in decreased levels of SERCA1, SERCA2 and RyR1 proteins, while DHPR, MyHC, α-sarcoglycan and actin protein levels remained unchanged (Fig. 2b, Fig. S2).

We sought to determine if this reduction of SERCA1/2 protein levels was also translated into deficient Ca^2+^ clearance capacity in human myotubes. Thus, we analysed SERCA specific ATPase activity in homogenates from LHCN-M2 myotubes and found a significant SERCA activity decline in CAPN3 knockdown myotubes (4.23 ± 1.55 mm ATP/min/mg protein) compared with NS controls (7.81 ± 1.25 mm ATP/min/mg protein; *P* < 0.05, Fig. 2C). We also measured intracellular Ca^2+^ levels in myotubes loaded with Fura2-AM and we found that in LHCN-M2 human myotubes, basal intracellular Ca^2+^ concentration is significantly increased in CAPN3 knockdown myotubes compared with NS controls (*P* < 0.05, *n* = 10, Fig. 2D). Also, when myotubes were stimulated with 130 mm KCl in the absence of extracellular Ca^2+^, we found that the Ca^2+^ clearance time is significantly increased in CAPN3-deficient myotubes (7.34 ± 0.83 s) compared with NS controls (5.72 ± 0.29 s; *P* ≤ 0.05, Fig. 2e and f). Taken together, our results indicate that CAPN3 knockdown myotubes display reduced SERCA1/2 protein levels and Ca^2+^ clearance capacity compared to controls, which is likely related with a higher basal intracellular Ca^2+^ concentration observed in CAPN3-deficient human myotubes.

Interestingly, silencing CAPN3 results in higher resting cytosolic [Ca^2+^] in human but not in mouse myotubes. Consistently, a previous study using primary mouse myotubes found reduced SR calcium content but normal resting intracellular Ca^2+^ levels in Capn3-deficient myotubes (Ref. 3). Differences in the resting Ca^2+^ levels observed between CAPN3-deficient human and mouse myotubes may be related with dissimilar Ca^2+^ buffer capacity (Ref. 5). Indeed, parvalbumin, which is a major cytosolic Ca^2+^ buffer in the mouse skeletal muscle, is expressed at very low levels in human muscles (Ref. 18). In this line, while parvalbumin was not detected in human LHCN-M2 myotubes by western blot, we found that Capn3-deficient mouse C2C12 myotubes showed significantly higher parvalbumin levels compared with control myotubes (174.35 ± 13.10% versus 100 ± 6.01%, *P* < 0.05; Fig. S9). Therefore, in Capn3-deficient mouse myotubes, the higher parvalbumin expression could be buffering cytosolic calcium increases resulting from SERCA1/2 deficiency.

### SERCA levels are reduced in CAPN3 deficient human muscles

**Figure 3.**
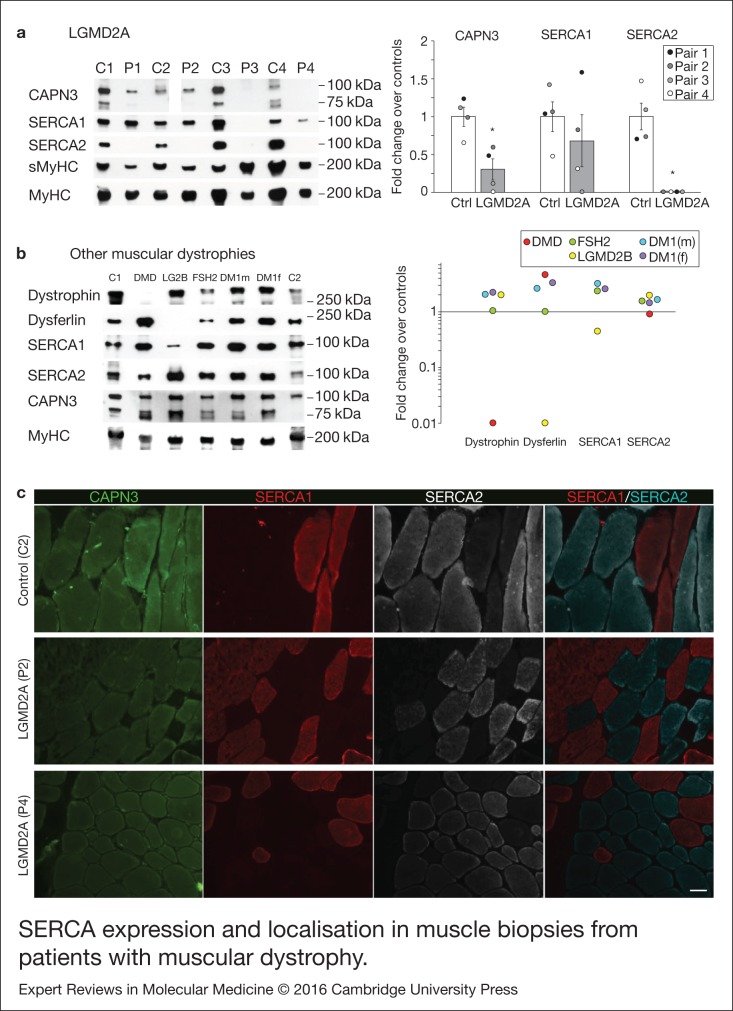
SERCA expression and localisation in muscle biopsies from patients with muscular dystrophy. (a) Analysis of CAPN3 (SPA), SERCA1, SERCA2, slow (sMyHC) and total myosin heavy chain (MyHC) expression in muscle samples from 4 LGMD2A patients (P1–4) and 4 controls (C1–C4). All muscles from LGMD2A patients show absence of SERCA2 protein. Left panel shows representative western blot signals. Right panel depicts optical density values of proteins normalised to MyHC in LGMD2A patients and expressed as fold change over controls. *P < 0.01 versus control samples. Statistical significance was determined using unpaired, 2-tailed Student's *t* test. (b) Western blot analysis of dystrophin, dysferlin, SERCA1, SERCA2, CAPN3 and MyHC in two controls (C1-C2) and 5 patients with other muscular dystrophies: fascioscapulohumeral muscular dystrophy (FSH2), Duchenne (DMD), LGMD2B (LG2B); myotonic dystrophy (DM1). Baseline represents average of control sample levels C1 and C2. None of these dystrophic patients show deficient expression of SERCA2 in the muscle. (c) Cross-sections from 1 control (C2) and 2 LGMD2A (P2, P4) human muscles co-immunostained for CAPN3, SERCA1 and SERCA2. Immunofluorescence analysis showed reduced expression of CAPN3 in LGMD2A muscle fibres. SERCA2 levels appear reduced in the LGMD2A samples and showed a preferential localisation near the sarcolemma. Scale bar: 50 µm.

We analysed expression of SERCA1/2 proteins in human skeletal muscle samples from control and LGMD2A dystrophic patients, in order to determine if our results obtained in vitro were relevant for this disease. For this analysis we used muscle biopsies from 4 LGMD2A patients (P1–4) and 4 sex- and age-matched controls (C1–4). Clinical information of control and dystrophic patients used in this study are shown in Table 1. Western blot analysis showed that the four LGMD2A patients analysed had significantly lower amount of CAPN3 protein compared with control samples, when normalised with total myosin heavy chain (MyHC) (30 ± 14%; *P* < 0.05, Fig. 3a). SERCA1 protein levels were not significantly different in control versus LGMD2A samples, although P3 and P4 samples, which had the lowest CAPN3 levels among LGMD2A samples, showed a distinct reduction of SERCA1 levels compared with control samples (P3 = 1%; P4 = 31%). On the other hand, SERCA2 protein was undetectable in all the LGMD2A samples, while it was normally expressed in control samples. Absence of SERCA2 in LGMD2A samples was not because of lower numbers of slow muscle fibres in these samples, as demonstrated by the similar levels of slow myosin heavy chain (sMyHC) observed in control and LGMD2A samples (Fig. 3a).

Next, we sought to analyse SERCA2 expression in skeletal muscles from patients with other forms of muscular dystrophy. For this analysis, we used muscle biopsies from patients with Duchenne muscular dystrophy (DMD), LGMD2B muscular dystrophy (LG2B), facioscapulohumeral muscular dystrophy (FSH2) and myotonic dystrophy type 1 (DM1; Table 1). As expected, we found that dystrophin and dysferlin protein levels were absent in DMD and LGMD2B samples, respectively (Fig. 3b). However, we did not find any reduction of SERCA2 levels in any of these dystrophic samples. Likewise, these dystrophic samples expressed normal levels of SERCA1, except for the LGMD2B sample, which showed a 55% decrease compared to controls levels. None of these dystrophic samples showed secondary reduction of CAPN3 levels.

We next performed immunofluorescence analysis of CAPN3 and SERCA proteins in one control (C2) and 2 LGMD2A samples (P2, P4) (Fig. 3c, Fig. S10). CAPN3 staining appeared reduced in both LGMD2A patients and, while SERCA proteins were detected in these patients, SERCA2 expression seemed reduced and preferentially localised at the sarcolemma. Interestingly, in the LGMD2A samples, a fraction of slow fibres abnormally expressed low levels of SERCA1, suggesting that SERCA1 may partially compensate for the reduction of SERCA2 (Fig. S10). These results are in line with the previous data obtained by western blot analysis, and altogether, our findings support a role of SERCA proteins in the pathophysiology of LGMD2A.

### CAPN3 deficiency disrupts small Ankyrin 1 (sAnk1) protein expression and localisation

**Figure 4.**
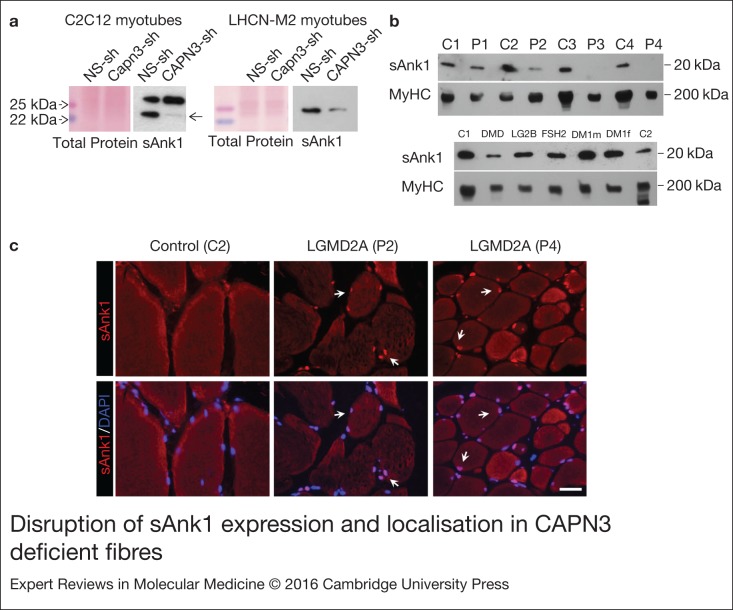
Disruption of sAnk1 expression and localisation in CAPN3-deficient fibres. (a) Representative Western blots showing reduced sAnk1 protein levels in mouse Capn3-deficient C2C12 myotubes (left) and human CAPN3-deficient LHCN-M2 myotubes (right) compared with controls. Membranes were stained with Ponceau-S for verification of equal total protein loaded. (b) Western blot analysis of sAnk1 expression in human muscle samples. (c) Cross sections from a control and two LGMD2A human muscles co-immunostained for sAnk1 and DAPI. Note the higher accumulation of sAnk1 at the nuclei in the LGMD2A fibres (arrows). Scale bar: 25 µm.

sAnk1, an SR protein, which binds with the giant sarcomeric proteins titin and obscurin, is essential for maintenance of the SR network integrity and its organisation around the contractile apparatus (Refs 19, 20). Silencing sAnk1 expression in rat myofibres results in reduced expression of SERCA1 (Ref. 20), which is similar to the effect observed in CAPN3 knockdown myotubes. Therefore, we sought to determine if sAnk1 expression was altered in the absence of CAPN3. For this purpose, we analysed expression of sAnk1 in CAPN3 knockdown myotubes, and found reduction of sAnk1 protein levels in both mouse and human CAPN3-deficient myotubes compared with NS controls (Fig. 4a). In this line, we found that in human muscle samples sAnk1 was strongly reduced in the LGMD2A samples with nondetectable CAPN3 expression (P3, P4; Fig. 4b, upper panel), while expression levels of sAnk1 in muscle samples from other forms of muscular dystrophy were comparable with control levels (Fig. 4b, lower panel). Using immunohistochemistry, we were able to detect expression of sAnk1 in P2 as well as in P4 LGMD2A samples. Interestingly, this analysis showed that in both LGMD2A patients, sAnk1 was abnormally accumulated at the nuclei (Fig. 4c). Taken together, our results indicate that in the skeletal muscle, CAPN3 may have an impact on sAnk1 expression levels and on its appropriate localisation to the SR. Further studies will be needed in order to determine the role of sAnk1 in the nucleus.

### CAPN3 interacts with SERCA1, SERCA2 and sAnk1 in the skeletal muscle

**Figure 5.**
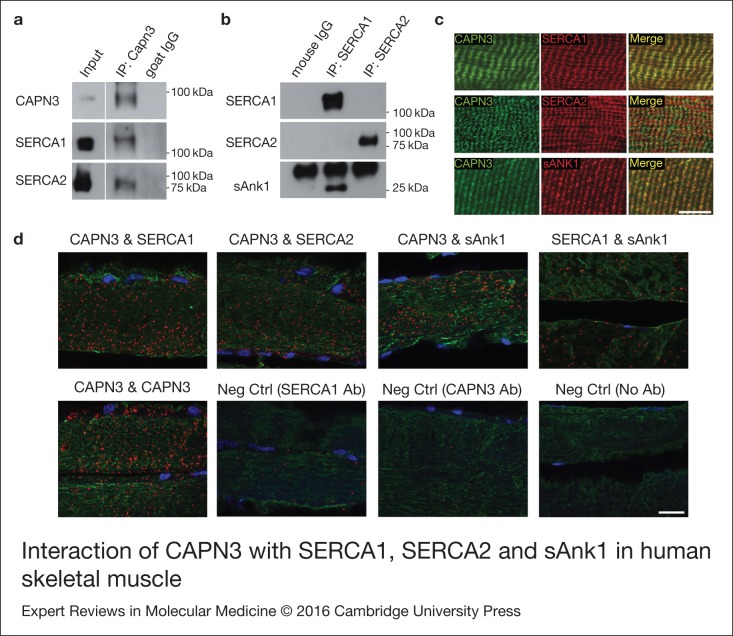
Interaction of CAPN3 with SERCA1, SERCA2 and sAnk1 in human skeletal muscle. (a) Immunoprecipitation (IP) of CAPN3 with a goat polyclonal antibody (pIS2C) in a *vastus lateralis* muscle from a healthy donor. Both, SERCA1 and SERCA2 are detected in the CAPN3 IP. White lines indicate noncontiguous lanes run on the same gel. Input: protein extract. (b) SERCA1 and SERCA2 were immunoprecipitated (IP) with specific monoclonal antibodies in the same muscle. sAnk1 is detected in SERCA1 IP but not in SERCA2 IP. (c) Longitudinal sections from a dorsal human muscle co-immunostained for CAPN3-SERCA1, CAPN3-SERCA2 and CAPN3-sAnk1, showing similar distribution pattern of CAPN3 (green), SERCA1 (red), SERCA2 (red) and sAnk1 (red) in the panels labelled “Merge”. Scale bar: 10 µm. (d) Co-localisation analysis of CAPN3, with SERCA1, SERCA2 and sAnk1 in longitudinal sections from human dorsal muscle using in situ PLA. Red spots represent protein complexes in close proximity (<40 nm). Sections were double-stained for myosin heavy chain (MF20, green). Neg Ctrl, negative control with just one or no primary antibodies. Scale bar: 20 µm.

In view of the similar localisation pattern of CAPN3, SERCA1, SERCA2 and sAnk1 around the Z-discs and M-lines (Refs 20, 21), we wanted to assess if these proteins interacted within the skeletal muscle. First, we performed co-immunoprecipitation assays using protein extracts from the *vastus lateralis* of a healthy human donor. Indeed, we found that both SERCA1 and SERCA2 proteins co-immunoprecipitated with CAPN3 (Fig. 5a), which is in line with previous studies showing interaction between SERCA1 and Capn3 (Ref. 8). Absence of cross-reactivity between SERCA1 and SERCA2 antibodies was demonstrated by specific detection of each isoform in the immunoprecipitates (Fig. 5b). Interestingly, we found that sAnk1 co-immunoprecipitated with SERCA1 but not with SERCA2 (Fig. 5b). Next, we examined the distribution of CAPN3, SERCA1, SERCA2 and sAnk1 in the human skeletal muscle by immunofluorescence and we observed a highly similar localisation pattern and a broad co-localisation of CAPN3 with SERCA1, SERCA2 and sAnk1 (Fig. 5c). To further validate interaction of these proteins, we used the in situ PLA, which reveals protein complexes (<40 nm distance) at a single molecule resolution within the cellular context (Ref. 22). This analysis showed that CAPN3 interacts with SERCA1, SERCA2 and sAnk1 in the human skeletal muscle (Fig. 5d). Also, co-localisation of SERCA1 and sAnk1 was observed in the human muscle. Overall, our results indicate that CAPN3 and SERCA1/2 proteins form molecular complexes within the skeletal muscle, with a differential contribution of sAnk1 to the complexes formed by SERCA1 or SERCA2.

Lastly, to identify potential binding sites between CAPN3 and SERCA1, we used the powerful protein–protein interactions online prediction tool PRISM 2.0 (http://cosbi.ku.edu.tr/prism/), which is able to perform accurate predictions and find critical residues at the protein–protein interfaces (Ref. 23). The analysis was performed starting from the Protein Data Bank (http://www.rcsb.org/pdb/home/home.do) files of CAPN3 penta EF-hand domain (PEF) (4okh) and SERCA1 (3tlm) to yield a preferred interaction structure (PRISM template interface: 1u5iAB) with a binding energy of −28.0 kcal/mol. The residue interaction pattern predicted 20 interface interactions (Fig. S11) involving 13 different residues of CAPN3 and 16 residues of SERCA1, which encompass three helices from each protein. Potential binding sites for SERCA2 could not be determined because of lack of its PDB structure. However, since SERCA1 and SERCA2 share a high homology, particularly within the specific regions where SERCA1 is predicted to interact with PEF domain of CAPN3, we presume that SERCA2 would have similar binding regions as the ones predicted for SERCA1.

### Increased SERCA1 and SERCA2 protein degradation in CAPN3-deficient myotubes

**Figure 6.**
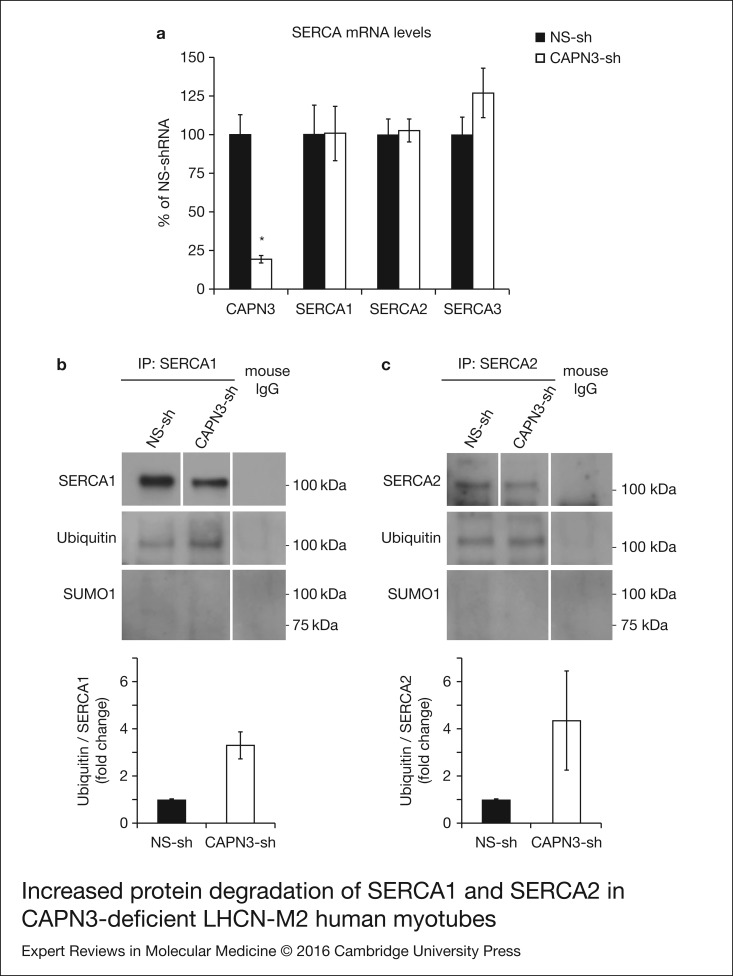
Increased protein degradation of SERCA1 and SERCA2 in CAPN3-deficient LHCN-M2 human myotubes. (a) CAPN3 and SERCA mRNA levels were analysed in human myotubes by quantitative RT–PCR. Statistical analysis showed significant decrease in CAPN3 expression (19.3 ± 2.6%) in CAPN3 knockdown myotubes as compared to matched controls (100 ± 12.8%). *N* = 3; *P < 0.005. No significant changes were observed in SERCA1, SERCA2 or SERCA3 mRNA expression levels. (b) SERCA1 and (c) SERCA2 ubiquitination and sumoylation were analysed in control and CAPN3-deficient human myotubes through SERCA1/2 immunoprecipitation with specific mouse monoclonal antibodies. Pools of control and CAPN3-deficient cultures were used for immunoprecipitation assays. *N* = 2 and *N* = 3 independent experiments were performed for SERCA1 and SERCA2, respectively. White lines indicate noncontiguous lanes run on the same gel. Ubiquitination of SERCA1 and SERCA2 in CAPN3-deficient myotubes was increased 3.30 and 4.35-fold, respectively, compared with controls. No SUMO1 specific sumoylation of SERCA1 and SERCA2 proteins was detected in controls or CAPN3-deficient myotubes.

Next, we sought to determine if CAPN3 deficiency affected transcription of ATP2A1 and ATP2A2 genes using real-time quantitative RT–PCR. Interestingly, we found that mRNA levels of SERCA1, SERCA2 and SERCA3 in CAPN3-deficient myotubes were comparable with mRNA expression in control samples, while CAPN3 mRNA levels were significantly reduced to 20% of control levels (Fig. 6a). This indicates that the reduced SERCA protein levels found in CAPN3-deficient myotubes are most likely because of protein degradation.

We hypothesised that SERCA proteins might be abnormally degraded by the ubiquitin–proteasome system, since CAPN3 deficiency has been previously associated with increased protein ubiquitination (Ref. 24). On the other hand, SUMO1 can be conjugated to lysine residues in SERCA2 and prevent its ubiquitination and degradation (Ref. 25). Therefore, we decided to analyse ubiquitination and sumoylation of SERCA1 and SERCA2 in control and CAPN3-deficient human myotubes. Our analysis revealed that in CAPN3-deficient myotubes, SERCA1 and SERCA2 ubiquitination is increased ~3- and 4-fold, respectively; whereas SERCA1/2 sumoylation was undetected in neither control nor CAPN3-deficient myotubes (Fig. 6b and c and Fig. S6).

## Discussion

We have demonstrated that silencing CAPN3 expression in mouse and human myotubes leads to a decrease in SERCA activity because of reduced SERCA1 and SERCA2 protein levels, the main forms differentially expressed in fast and slow muscle fibres, respectively (Refs 9, 10). This effect is not because of a deficit in cell maturation, since evaluation of overall myotube morphology and maturation markers such as DHPR and MyHC remained similar in CAPN3 knockdown and control myotubes. This is in contrast with a previous report that suggests involvement of CAPN3 in C2C12 differentiation (Ref. 26). This discrepancy may be because of the differences in the models used and/or the CAPN3 knockdown protocol.

Our results support that this reduction is because of protein degradation, since similar SERCA1 and SERCA2 mRNA levels were observed in CAPN3-deficient human myotubes. Reduced expression of other Ca^2+^-handling proteins, such as RyR1 and CamKII has been previously described in Capn3-deficient myofibres as well as in LGMD2A muscle biopsies (Refs 4, 6). However, to our best knowledge, this is the first evidence of modulation of SERCA1/2 protein levels by CAPN3.

Consistent with our in vitro results, analyses of human skeletal muscles show that SERCA1 and SERCA2 protein levels are reduced in the absence of CAPN3. However, while SERCA1 expression is solely reduced in LGMD2A samples with nondetectable levels of CAPN3, SERCA2 protein is patently reduced in all the LGMD2A samples analysed. Previous studies indicate that slow muscle fibres are predominantly affected in LGMD2A muscular dystrophy (Ref. 6). Given that SERCA2 is the major SERCA isoform in these fibres, our results suggest that in LGMD2A the higher susceptibility of slow fibres is caused by a loss of SERCA2 protein secondary to CAPN3 deficiency. In this regard, we have not detected any significant difference in the levels of sMyHC between control and LGMD2A muscles, suggesting that SERCA2 deficiency would precede the loss of slow muscle fibres in LGMD2A patients. Moreover, loss of SERCA2 protein seems to be specific of CAPN3 deficiency, since it is not observed in several patients with other forms of muscular dystrophy. However, given that reduction of CAPN3 expression has been reported in muscular dystrophies other than LGMD2A, we anticipate that these patients could also display reduced SERCA1/2 protein levels (Refs 27, 28, 29). A broader analysis of muscular dystrophic samples will address the diagnostic value of these changes. In this regards, diagnosis of LGMD2A patients based on classical phenotype and CAPN3 Western blot analysis is often inconclusive, since in LGMD2A patients, mutations in the CAPN3 gene do not always result in reduced CAPN3 protein levels (Ref. 30). Therefore, our results support that analysing SERCA2 expression levels in muscle biopsies could be relevant for diagnostic purposes as a potential indicator of CAPN3 deficiency, both primary and secondary.

Most interestingly, our results reflect that SERCA1 and SERCA2 could be used as targets in therapeutic strategies for muscular dystrophies showing CAPN3 deficiency. In this line, recent studies using mouse models of muscular dystrophies affecting dystrophin–glycoprotein complex have shown that increasing SERCA activity either by inducing HSP72 expression (Ref. 13), or through SERCA1 and SERCA2 overexpression using transgenic mice and adeno-associated virus gene therapy (Ref. 17) results in reduced muscle degeneration in these animals. The first clinical trial of SERCA2 restoration has already been conducted in patients with heart failure using adeno-associated virus gene therapy with promising results in terms of safety and disease markers (Ref. 31). In addition, in the past years a number of drugs that increase SERCA2 expression or function have been tested in animal models of heart failure, such as angiotensin and adrenoreceptor blockers, adrenergic agonists, hormones, glucocorticoids and natural antioxidant agents (Refs 5, 32). Alternatively, a recent study has reported functional rescue of a mutated SERCA1 by inhibiting its degradation through the ubiquitin–proteasome system in a cellular model of Brody disease, a skeletal muscle disorder caused by SERCA1 deficiency (Ref. 33). Our findings indicate that these strategies should also be considered as potential treatments for LGMD2A muscular dystrophy.

Studies performed with knockout mice have confirmed a major role of SERCA1 and SERCA2 in excitation–contraction coupling and muscle relaxation (Refs 34, 35). In humans, deficient activity of SERCA1 because of *ATP2A1* gene mutations causes a rare myopathy named Brody disease (Refs 36, 37, 38). In contrast, mutations in gene-encoding SERCA2 have only been associated with the dermatological Dariers's disease and with neuropsychiatric disorders (Ref. 39). However, these mutations affect the heart and skeletal muscle SERCA2a isoform as well as the SERCA2b isoform, which is the major SERCA isoform in nonmuscle tissues. The fact that no muscle phenotype has been described in patients with SERCA2 mutations could be because of compensatory mechanisms taking place in heart and slow muscle. Also, low levels of other SERCA isoforms, such as SERCA3 could further compensate for SERCA2 deficiency. Brody disease patients are characterised by myalgia, muscle cramps and exercised-induced stiffness (Ref. 38). Some LGMD2A patients display metabolic features such as exercised-induced myalgia or cramps (Ref. 40), which may be indicative of a SERCA deficiency. Therefore, a thorough evaluation of each LGMD2A patient clinical history may prove useful as an indicator of potentially defective SERCA function, which could be specifically treated.

**Figure 7.**
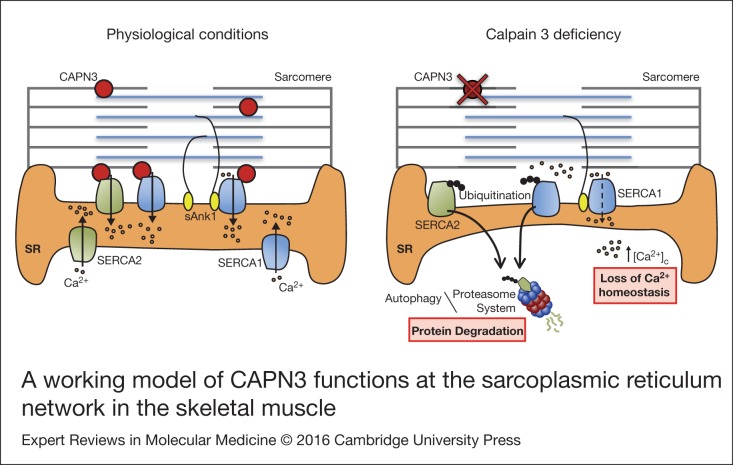
A working model of CAPN3 functions at the sarcoplasmic reticulum network in the skeletal muscle. Under physiological conditions, CAPN3 stabilises SERCA1 and SERCA2 protein complexes at the sarcoplasmic reticulum network. However, CAPN3 deficiency induces SERCA protein ubiquitination and degradation, and results in a loss of calcium homeostasis. Binding of sAnk1 to SERCA1 may help stabilise SERCA1 protein complexes under pathological conditions.

In search for pathways affected by CAPN3 deficiency that may be related with a decrease in SERCA proteins, we found that levels of sAnk1 are reduced in CAPN3-deficient muscles and myotubes. Also, abnormal nuclear localisation of sAnk1 was observed in CAPN3-deficient muscles. sAnk1 is an integral protein of the SR network that, like CAPN3, concentrates around Z-discs and M-lines (Ref. 41). This protein has been shown to interact with the giant sarcomeric proteins titin and obscurin, thus, connecting the SR with the contractile apparatus (Refs 42, 43). Since sAnk1 is essential for SERCA expression and localisation as well as for the integrity of the SR network compartment, reduction of sAnk1 levels observed in CAPN3-deficient fibres might be indicative of a disrupted SR architecture and SR-related proteins such as SERCA (Refs 19, 20). Co-immunoprecipitation analysis in human muscles revealed interaction between CAPN3 and the SR proteins SERCA1, SERCA2 and sAnk1. We propose that under physiological conditions, CAPN3 stabilises SERCA1 and SERCA2 protein complexes at the SR network. In LGMD2A, CAPN3 deficiency induces ubiquitination and degradation of SERCA proteins and ultimately results in a loss of calcium homeostasis. Binding of sAnk1 to SERCA1 may help stabilise SERCA1 protein complexes under pathological conditions, when CAPN3 levels are moderately reduced (Fig. 7).

Ubiquitination may target proteins to degradation through the ubiquitin proteasome system as well as through selective macroautophagy. Thus, the higher SERCA1 and SERCA2 ubiquitination levels observed in CAPN3-deficient myotubes may support an increased turnover through either the ubiquitin–proteasome system or the autophagic-lysosomal pathway. In this line, while several studies have demonstrated involvement of the ubiquitin proteasome pathway in the degradation of SERCA proteins in different models (Refs 33, 44, 45) there is also some evidence of degradation of specific SERCA proteins in a proteasome-independent manner. In particular, higher degradation of SERCA1 and SERCA2b proteins has been observed upon treatment with the proteasome inhibitor lactacystin (Refs 44, 45). This may be indicative of protein degradation through the autophagic-lysosomal pathway, since ubiquitin–proteasome inhibition is well known to induce autophagy. In this line, a recent study in LGMD2A patients supports proteasomal degradation as the main inductor of muscle atrophy in LGMD2A (Ref. 46). However, in these patients autophagy also seems to play a certain role, as shown by activation of markers related to autophagic-lysosomal pathway in LGMD2A muscles. In any case, further studies are needed in order to elucidate the specific mechanism by which CAPN3 deficiency induces acceleration of SERCA degradation, as this would open new avenues in the search for treatments against LGMD2A muscular dystrophy.

Previous studies have shown that CAPN3 localises to the SR and interacts with other SR proteins such as RyR1 and calsequestrin, with a structural rather than a proteolytic function (Refs 4, 6, 8). Adding to these studies, our findings support a relevant feature of CAPN3 as a multifunctional protein with different roles at specific locations and, within the SR, a crucial function stabilising SR-related proteins, such as SERCAs, Ank1 and RyR1 (Refs 4, 6, 8). In the future, novel CAPN3 functions will be likely discovered as we further our knowledge of CAPN3-binding partners and signalling pathways.

In conclusion, we have generated new evidence of the impact of CAPN3 deficiency on the Ca^2+^-mediated pathological mechanism of LGMD2A and on the stability of essential SR proteins such as SERCA1, SERCA2 and sAnk1. Although further experiments are required in order to address the specific contribution of SERCA towards muscle degeneration in LGMD2A, this study constitutes a reasonable foundation for the development of therapeutic approaches for LGMD2A that target SERCA2, SERCA1 or sAnk1.

## Supplementary material

For supplementary material accompanying this paper visit http://dx.doi.org/10.1017/erm.2016.9. click here to view supplementary material
