# Supplementary material for: Calpain 3 deficiency affects SERCA expression and function in the skeletal muscle
Source: Expert Rev Mol Med. 2016 Apr 8;18:e7. doi: 10.1017/erm.2016.9 (PMC4836212; doi:10.1017/erm.2016.9)
Supplement: Supplementary file 1 [file S1462399416000090sup001.pdf]

Figure S1

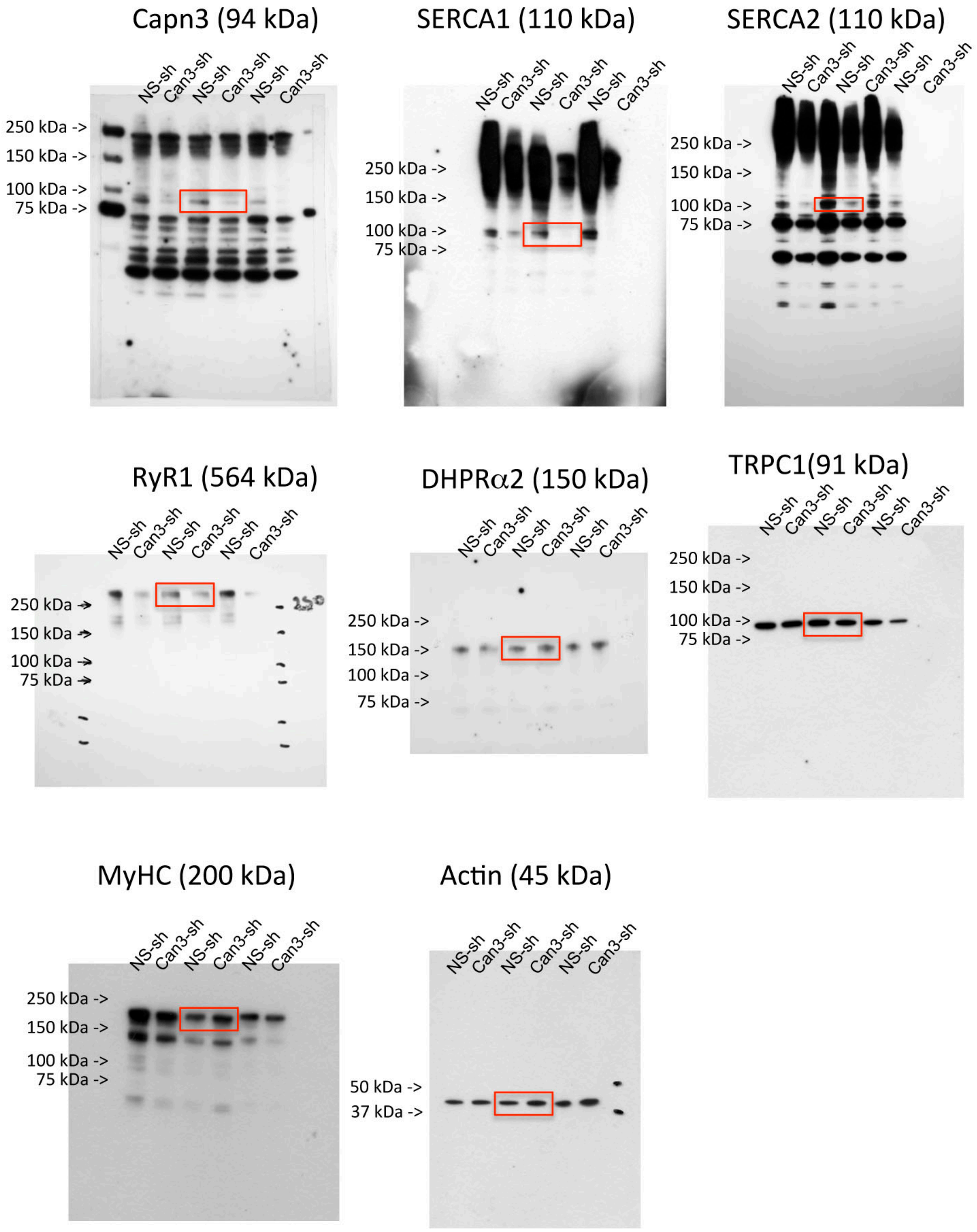

Figure S1. Uncropped western blots from Figure 1. Red rectangles depict selected areas shown in the original Figure

Figure S2

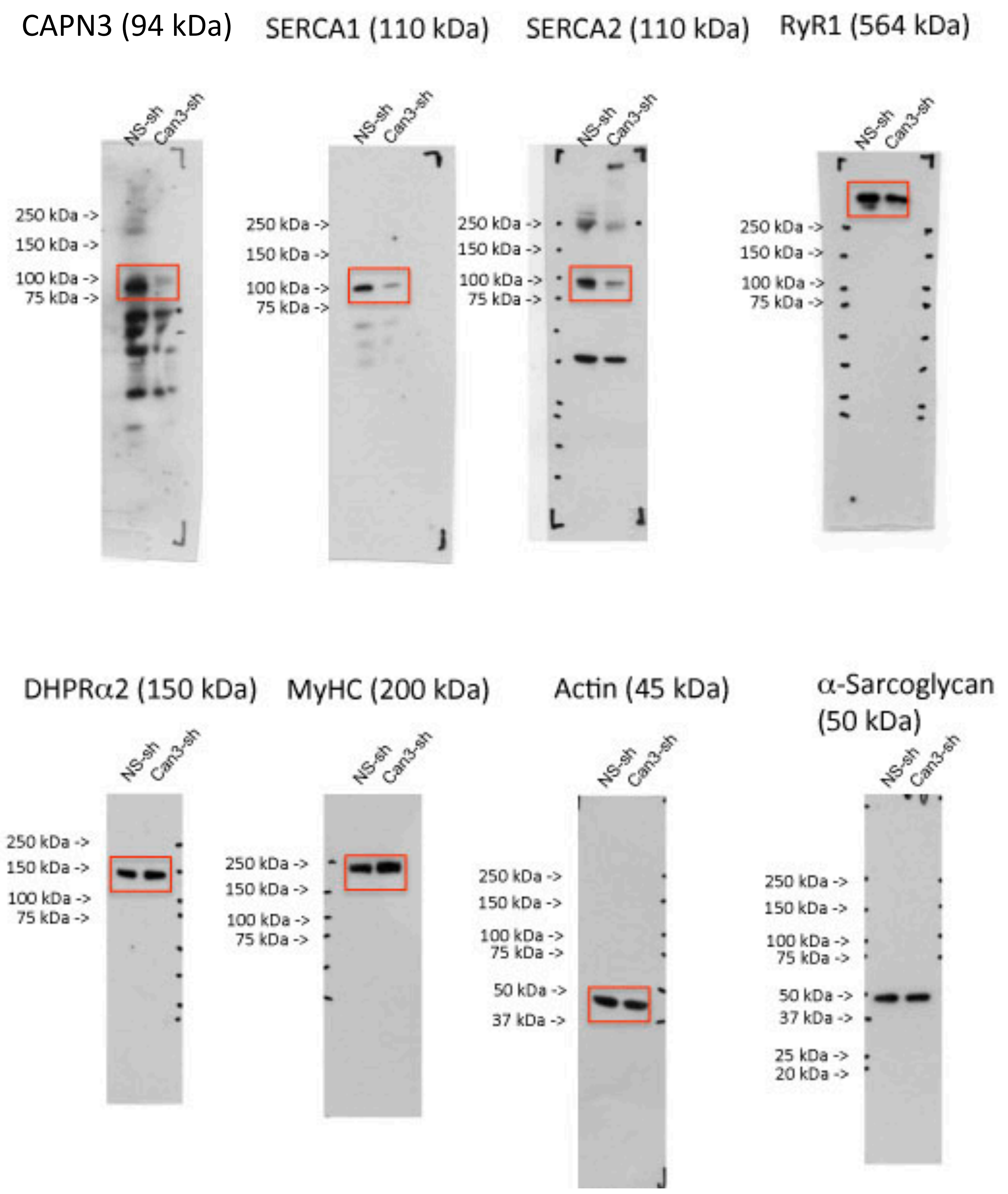

Figure S2. Uncropped western blots from Figure 2. Red rectangles depict selected areas shown in the original Figure. A representative western blot of α-sarcoglycan is included, showing that α-sarcoglycan levels remain unaltered in CAPN3-sh treated myotubes compared to NS-sh controls.

Figure S3

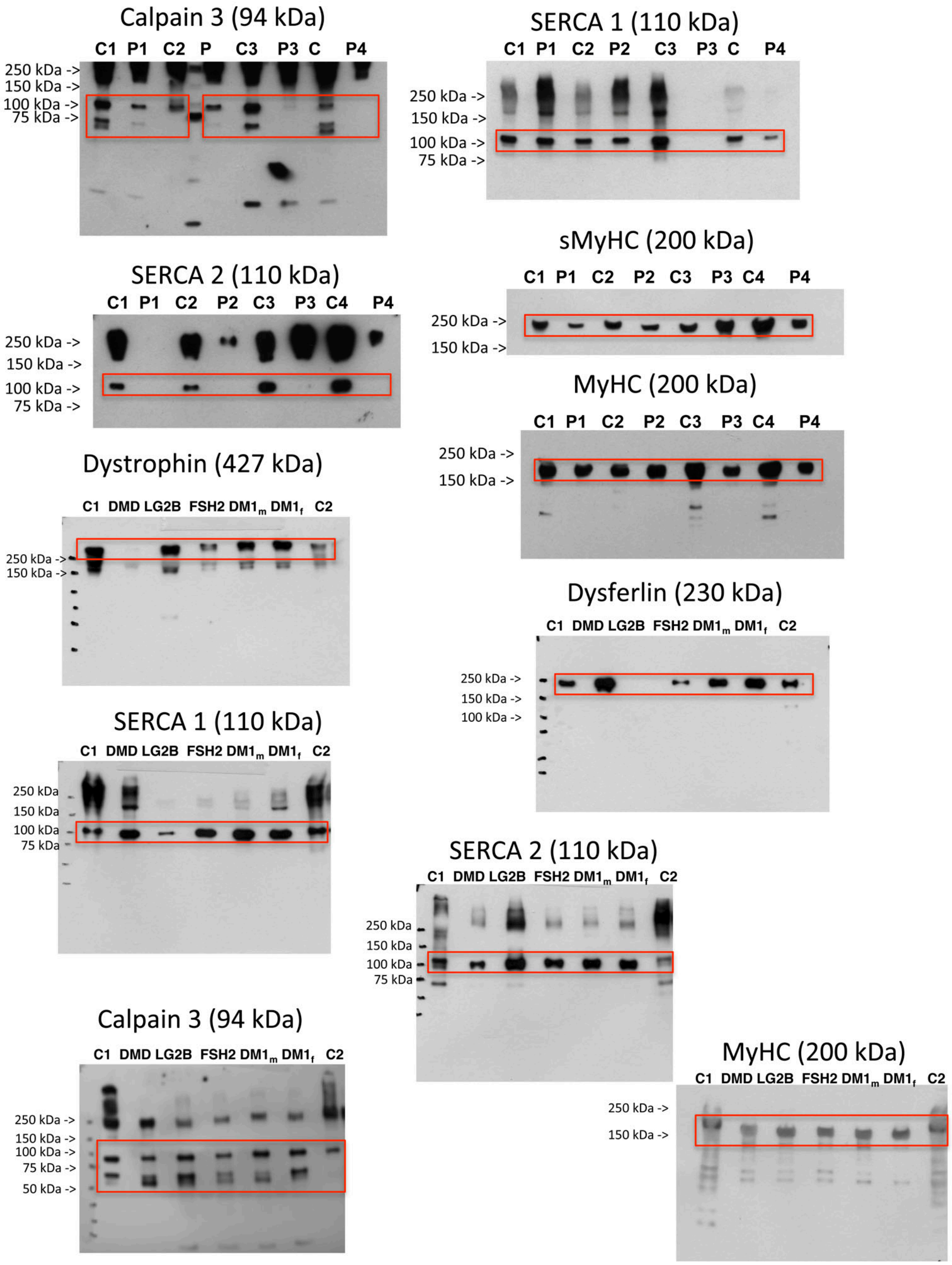

Figure S3. Uncropped western blots from Figure 3. Red rectangles depict selected areas shown in the original Figure.

Figure S4

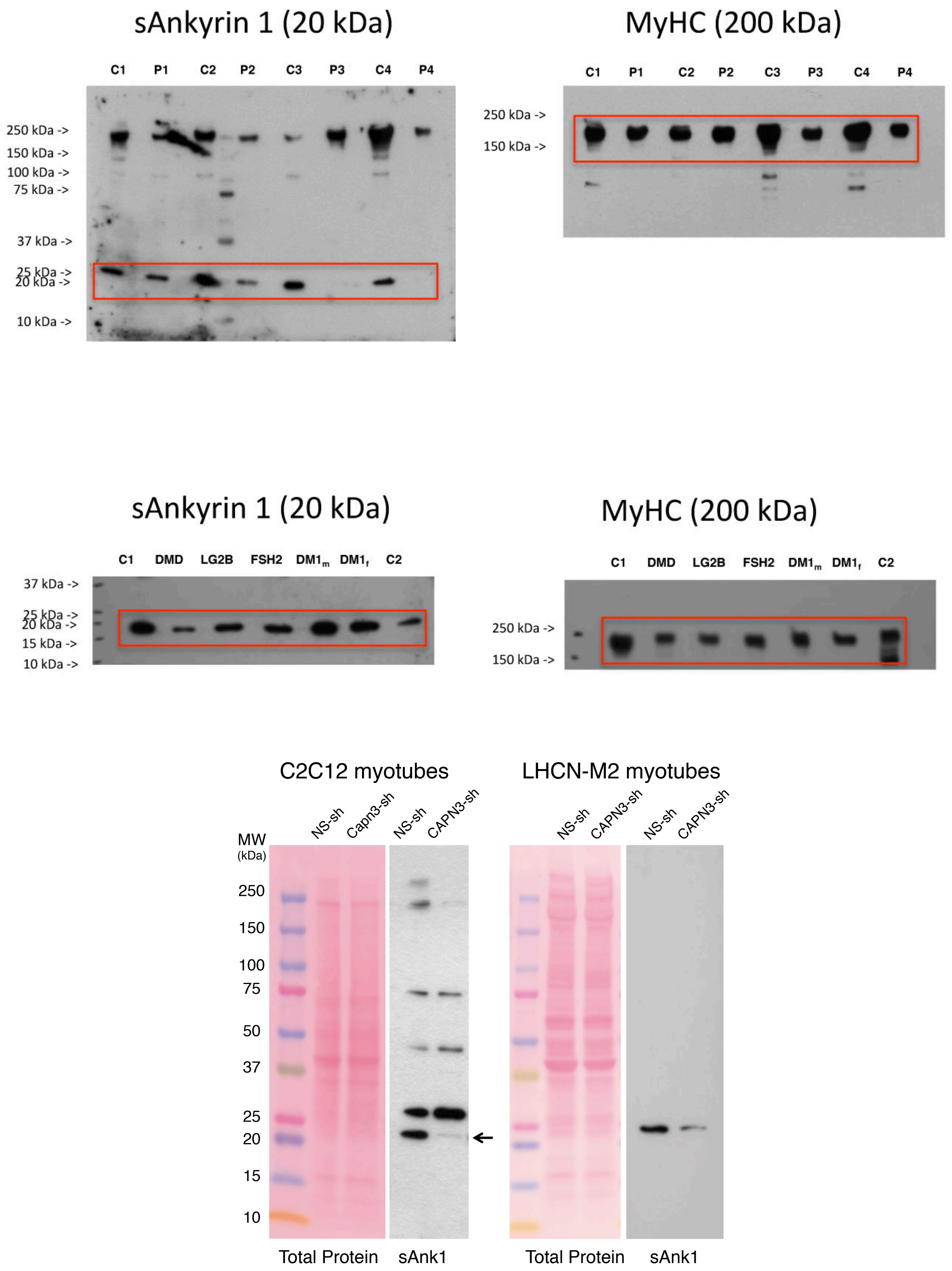

Figure S4. Uncropped western blots from Figure 4. Red rectangles depict selected areas shown in the original Figure.

Figure S5

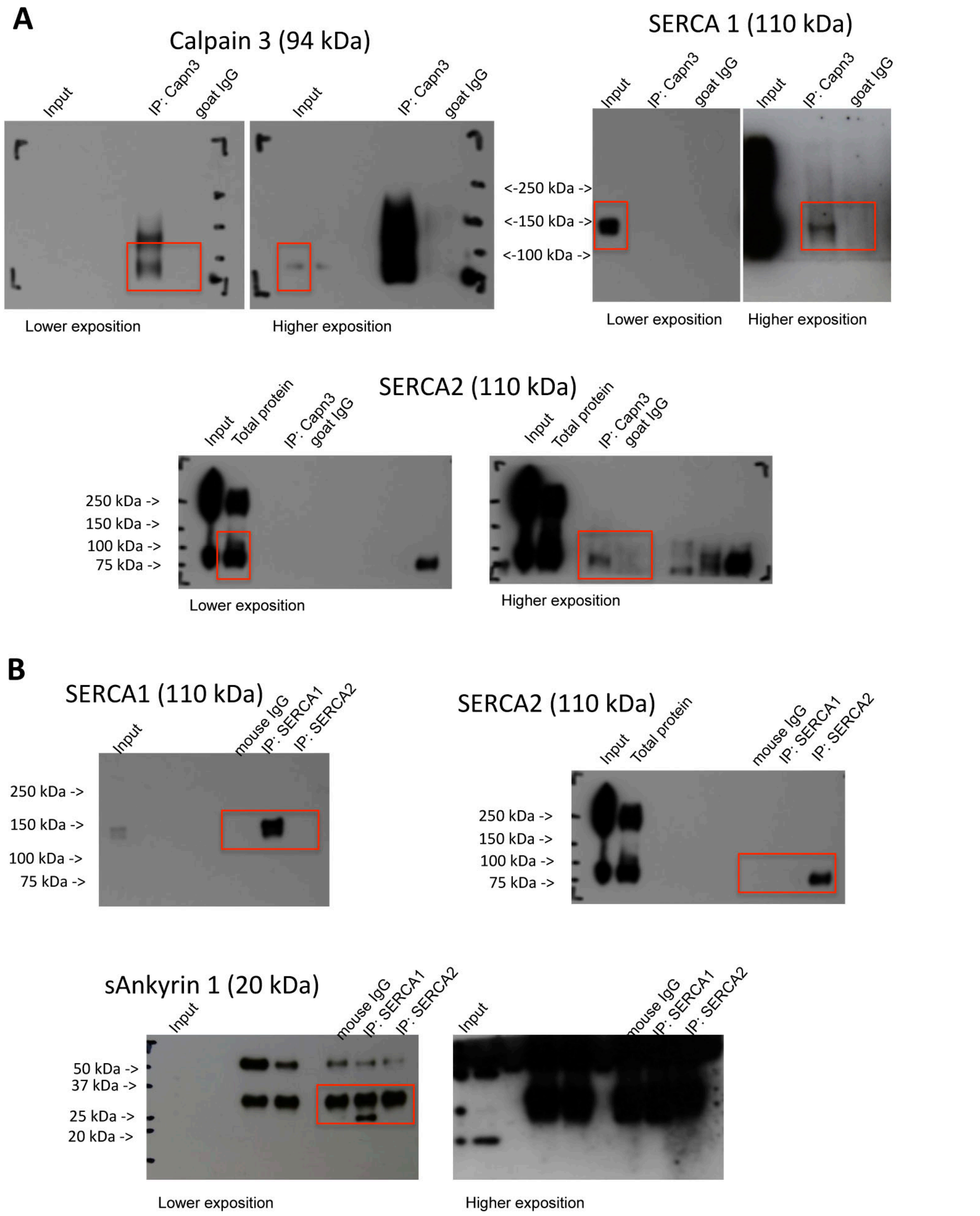

Figure S5. Uncropped western blots from Figure 5. Red rectangles depict selected areas shown in the original Figure.

Figure S6

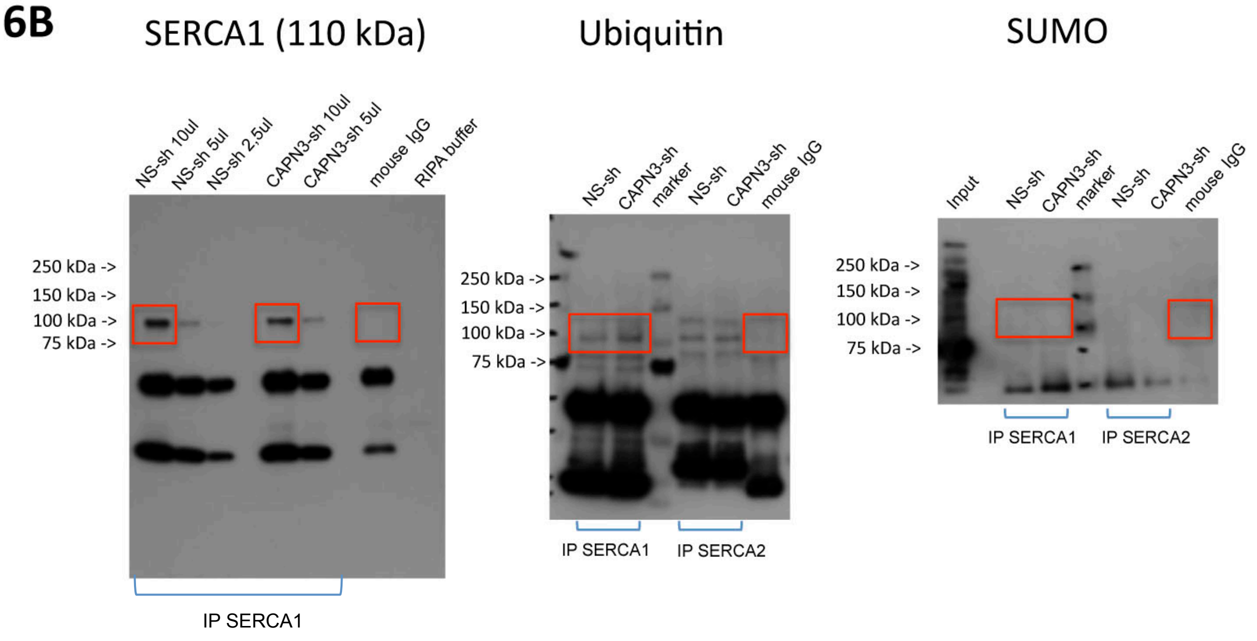

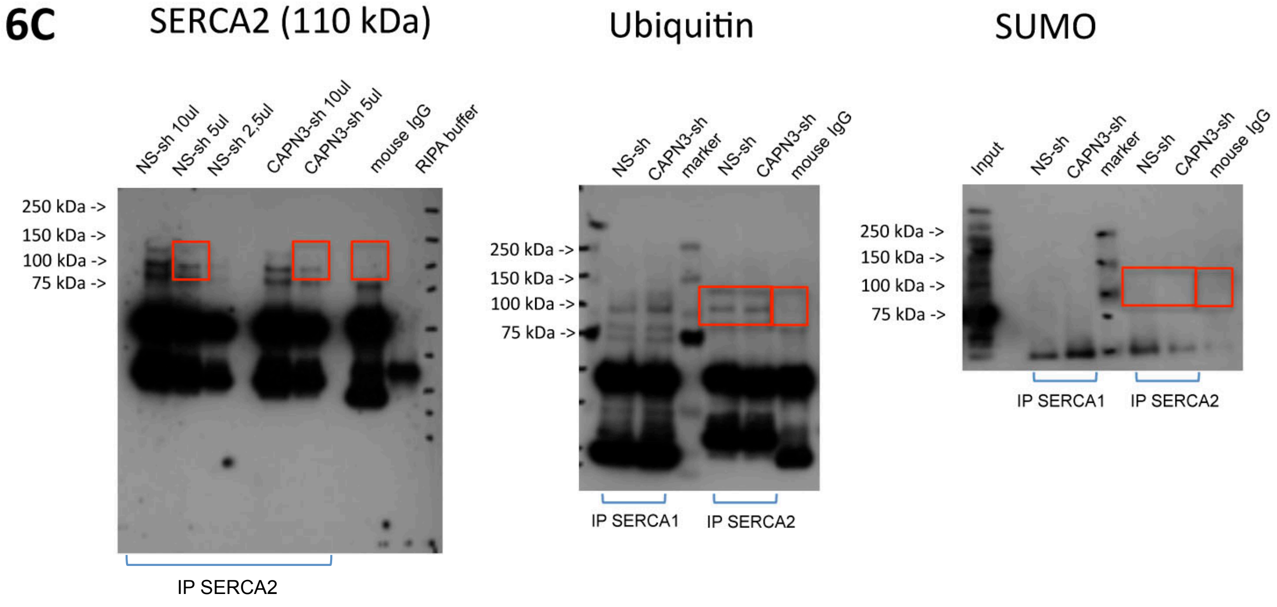

Figure S6. Uncropped western blots from Figure 6. Red rectangles depict selected areas shown in the original Figure.

Figure S7

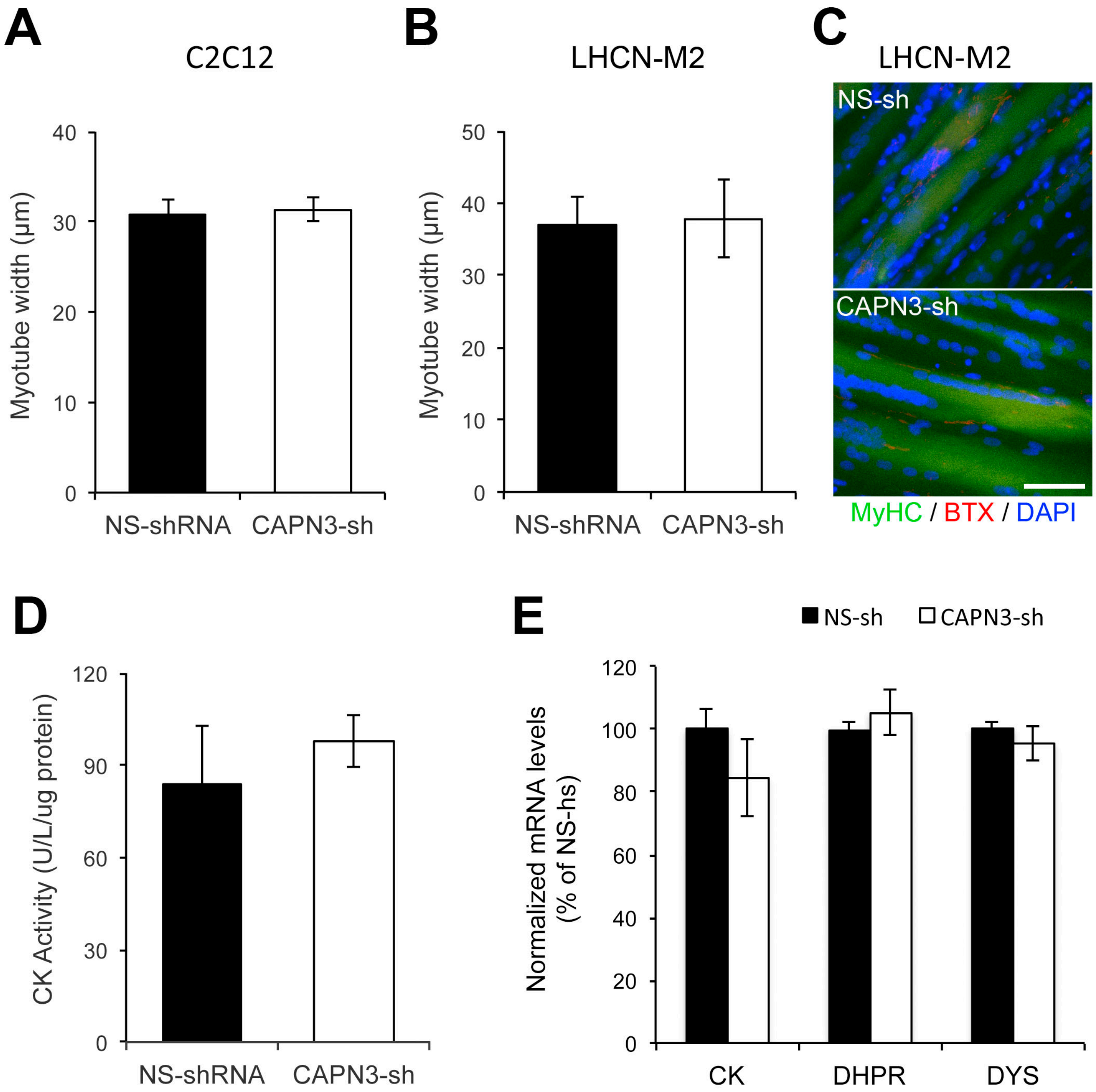

Figure S7. Similar maturation levels in C2C12 and LHCN-M2 were observed in control (NS-shRNA) and calpain 3-deficient myotubes (Capn3-sh; CAPN3-sh), as determined by **(A, B)** quantification of myotube width, **(C)**, immunocytochemistry **(D)** creatine kinase activity, or **(E)** mRNA levels of creatine kinase (CK), dihydropyridine receptor (DHPR) and dystrophin (DYS). Assays in **B-E** were performed in human LHCN-M2 myotubes as described in the material and methods section. No significant differences were observed in any of these assays between CAPN3-deficient and control myotubes. **A, B)** Myotube width was determined in 10 representative myotubes from each culture. **C)** Immunocytochemistry of NS-shRNA and CAPN3-deficient myotubes show similar myotube maturation levels. Myosin heavy chain expression (MyHC), nuclei number and localization (DAPI), and aggregation of acetylcholine receptors (Texas red conjugated α-bungarotoxin, BTX) are commonly used markers of myotube maturation. Scale bar= 50 μm. **A-E)** Data are expressed as mean ± SEM of at least N=3 different experiments.

Figure S8

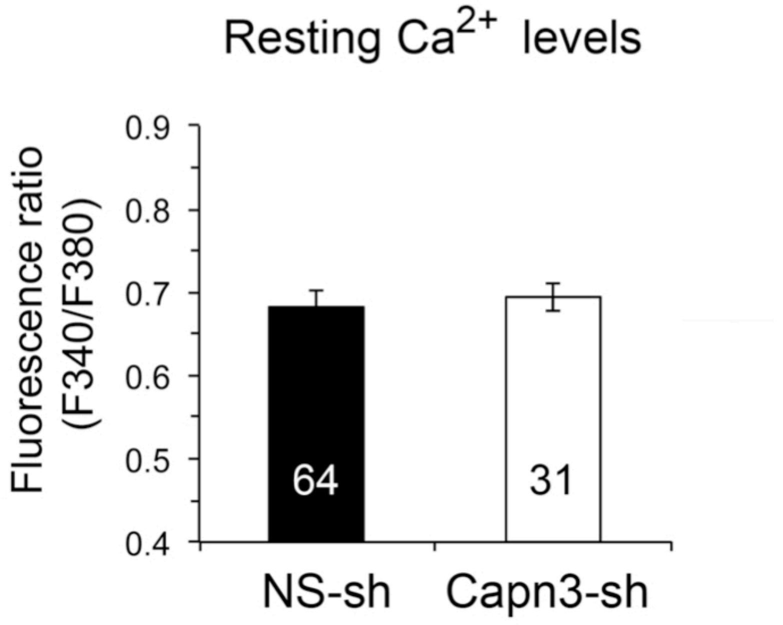

Figure S8. Resting cytosolic Ca2+ in C2C12 myotubes. Intracellular [Ca^{2+}] was measured with the ratiometric fluorochrome Fura-2AM in the presence of 2 mM extracellular Ca^{2+}. No difference was observed between control (NS-sh) and Capn3-deficient myotubes (Capn3-sh). Numbers of myotubes recorded are shown in the graph (n=6 experiments).

Figure S9

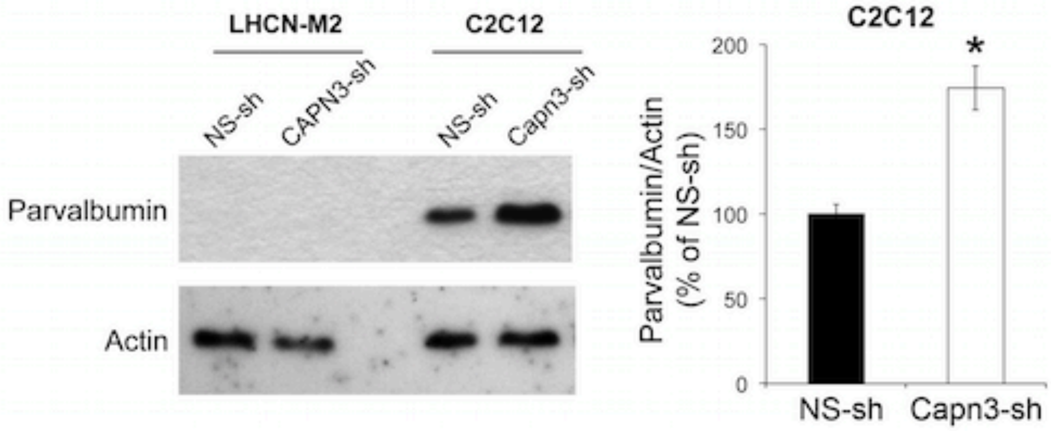

Figure S9. Parvalbumin expression in human LHCN-M2 and mouse C2C12 myotubes. Protein levels of parvalbumin were found increased in Capn3-shRNA mouse myotubes compared to NS controls (174 ± 13.10% vs. 100 ± 6.01%; n=3, *P<0.05). No parvalbumin was detected in human LHCN-M2.

Figure S10

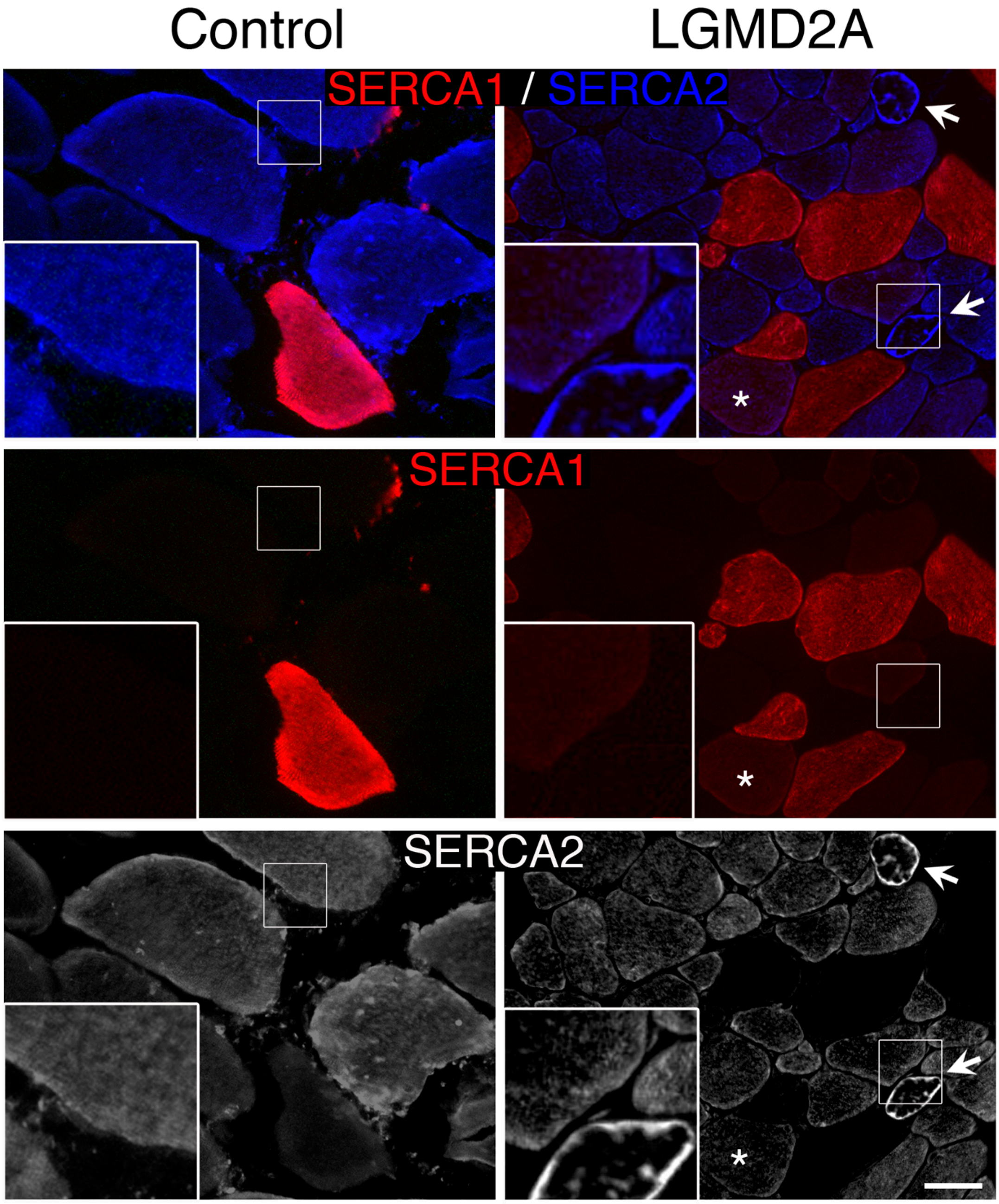

Figure S10. Cross sections of human muscles co-immunostained for SERCA1 and SERCA2. Overall SERCA2 levels appear reduced in the LGMD2 sample, mostly in the central region of the fibres. Note the higher accumulation of SERCA2 near the sarcolemma, in some fibres (arrows). A fraction of slow fibres of the LGMD2A patient co-express SERCA2 and low levels of SERCA1 (asterisks). Scale bar: 50 μm.

Figure S11

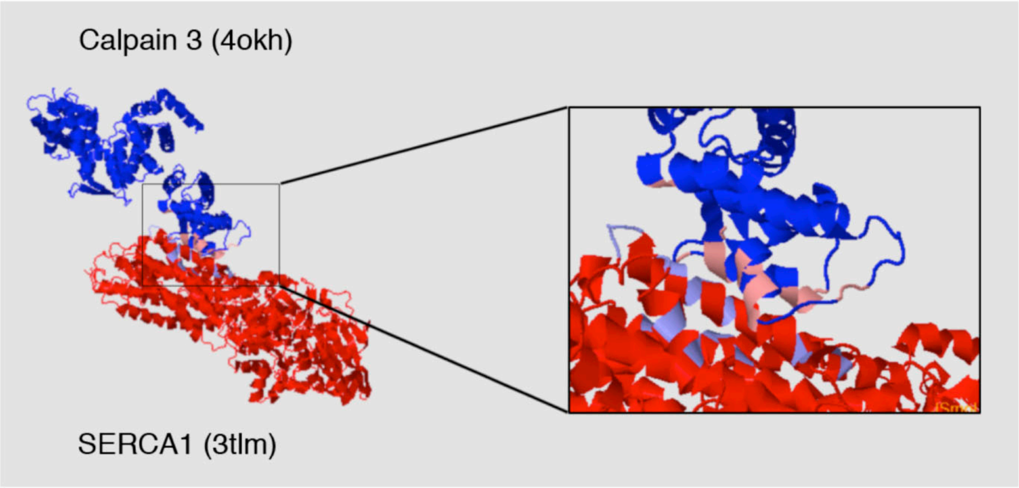

Interface Residues Contacts:

Calpain3		SERCA1
4okh_C_ILE_806	<-->	3tlm_A_TRP_927
4okh_C_VAL_811	<-->	3tlm_A_LEU_970
4okh_C_ILE_765	<-->	3tlm_A_LEU_801
4okh_C_MET_819	<-->	3tlm_A_ASP_962
4okh_C_GLN_761	<-->	3tlm_A_TRP_931
4okh_C_GLU_695	<-->	3tlm_A_ASP_950
4okh_C_GLU_695	<-->	3tlm_A_PRO_951
4okh_C_ARG_769	<-->	3tlm_A_SER_941
4okh_C_ILE_807	<-->	3tlm_A_TRP_927
4okh_C_LEU_815	<-->	3tlm_A_LEU_970
4okh_C_MET_768	<-->	3tlm_A_ILE_99
4okh_C_ASN_759	<-->	3tlm_A_TRP_931
4okh_C_ILE_765	<-->	3tlm_A_LEU_938
4okh_C_LYS_773	<-->	3tlm_A_PHE_92
4okh_C_ARG_769	<-->	3tlm_A_LEU_938
4okh_C_ARG_769	<-->	3tlm_A_LEU_942
4okh_C_MET_819	<-->	3tlm_A_PHE_944
4okh_C_LEU_812	<-->	3tlm_A_LEU_967
4okh_C_ASN_760	<-->	3tlm_A_TRP_107
4okh_C_GLU_695	<-->	3tlm_A_VAL_949

Figure S11. Prediction of protein-protein interaction between CAPN3 and SERCA1 using PRISM 2.0 webserver.
Upper panel shows protein data bank 3D structures of CAPN3 PEF domain (blue, 4okh) and SERCA1 (red, 3tlm) and their protein-protein interface. Lower panel lists the 20 interface residues contacts between CAPN3 and SERCA1. According to the PDB numbering, the following set of helices is involved in the interaction: SERCA1 helix 4 (3 residues), helix 40 (9 residues) and helix 42 (3 residues); CAPN3 helix 18 (2 residues), helix 21 (7 residues) and helix 23 (4 residues).

Table S1. Primer sequences used for real-time qPCR analysis on human LHCN-M2 myotubes.

	Forward Primer	Reverse Primer
**Calpain 3**	GAAAAGAGGAACCTCTCTGAGGAA	CGAAGATGATGGGCTTGGTT
**SERCA1**	TACGATGAGATCACAGCCATGAC	ATCCCATGGCAATGCCAAT
**SERCA2**	AAAGCTAAAGACATAGTTCCTGGTGAT	AGCAGGAACTTTGTCACCAACA
**SERCA3**	CGATACCTGGCTATCGGAGTGTAC	CAGGAAGTTCCTCAGCTGGTAGAA
**Creatine Kinase**	GAAGCTCTCTGTGGAAGCTCTCA	CCTTCTCCGTCATGCTCTTCA
**HPRT1**	CATGGACTAATTATGGACAGGACTGA	TGAGCACACAGAGGGCTACAA
**DHPRalpha1s**	GCCATCTCCGTGGTGAAGAT	CACTGCACCACGTGCTTCA
**Dystrophin**	ACAGGGCAAAAACTGCCAAA	CGCAGTGCCTTGTTGACATT
